# Health check attendance association with health and study-related factors: a register-based cohort study of Finnish university entrants

**DOI:** 10.1265/ehpm.22-00032

**Published:** 2022-08-19

**Authors:** Noora Seilo, Susanna Paldanius, Reija Autio, Kristina Kunttu, Minna Kaila

**Affiliations:** 1 Faculty of Medicine and Health Technology, Tampere University, Tampere, Finland; 2 Faculty of Social Sciences, Tampere University, Tampere, Finland; 3 Finnish Student Health Service, Finland; 4 Public Health Medicine University of Helsinki, Helsinki, Finland

**Keywords:** Health check, Student health care, Health behavior, Health promotion, Health services research

## Abstract

**Background:**

General health checks are an established component of preventive health care in many countries. Declining participation rates have raised concerns in health care providers. Understanding the reasons for attendance and non-attendance is necessary to improve the preventive health care system. The aim of this study was to examine health- and study-related factors associated with university entrants’ health check attendance.

**Methods:**

Since 2009, an electronic health questionnaire (eHQ) has been conducted yearly to all Finnish university entrants by the Finnish Student Health Service (FSHS) to screen students for a general health check. The questionnaire comprises 26 questions about health, health habits and studying. The study population consisted of the 3346 entrants from the 2011–2012 academic year who were referred to a health check based on their eHQ responses. The eHQ data were linked with health check attendance information. Multivariable logistic regression was used to study the associations between the questionnaire responses and non-attendance of the health check.

**Results:**

Male sex (OR 1.6, 95% CI % 1.4–1.9) and low engagement with studies (OR 1.5, 95% CI 1.2–2.0) were the variables most strongly associated with non-attendance. Having low state of mind was negatively associated with health check non-attendance thus enhanced the health-check attendance (OR 0.6, 95% CI 0.5–0.8).

**Conclusions:**

The results suggest that providing health checks in student health care may serve as a way of reaching students with health concerns. However, motivating males and smokers to attend general health checks continue to be a challenge also in a university student population. That low engagement with studies associates with health check non-attendance points to need to improve collaboration between universities and student health care.

**Supplementary information:**

The online version contains supplementary material available at https://doi.org/10.1265/ehpm.22-00032.

## Introduction

General health checks are an established component of preventive health care in many countries despite the inconclusive evidence of their effects [1, 2]. Declining attendance rates have been a challenge for health care providers [3].

In previous studies in adult populations, the reasons behind health check non-attendance have been diverse. In general, attenders have been found to be older than non-attenders [4]. Males and socioeconomically disadvantaged people have been less likely to attend [5, 6]. Of the health-related reasons, smoking, heavy drinking, physical inactivity, and obesity have been shown to be associated with non-attendance [5, 7]. Further, several health check system related reasons for non-attendance have been described, such as suitable timing and the location of the health check provider [8, 9].

Student health services of university students, including preventive and medical care, are provided nationally by the Finnish Student Health Service (FSHS) in Finland. As previously described in depth, the basis of preventive work at the FSHS is a statutory two-phased health examination process provided to all university entrants [10]. The process is targeted to detect risks for study ability early in studies [11] and consists of an electronic health questionnaire (eHQ), a screening tool, followed by a health check if needed [10–12]. Participation in the health examination process is voluntary and free of charge for students and it is their responsibility to make the health check appointment.

Health check attendance has been assessed in adult populations, however, in most cases young adults under 35 years have not been included. Specifically, there is lack of evidence about health check attendance of university students. Gaining information about the reasons behind the non-attendance of health checks is especially important in a society, where preventive services are publicly funded and the provision of health checks to university entrants is statutory.

The aim of this study was to describe factors associated with the health check attendance of university entrants. The specific research question was: How are university entrants’ responses to the eHQ questions associated with health check non-attendance.

## Methods

**Fig. 1:**
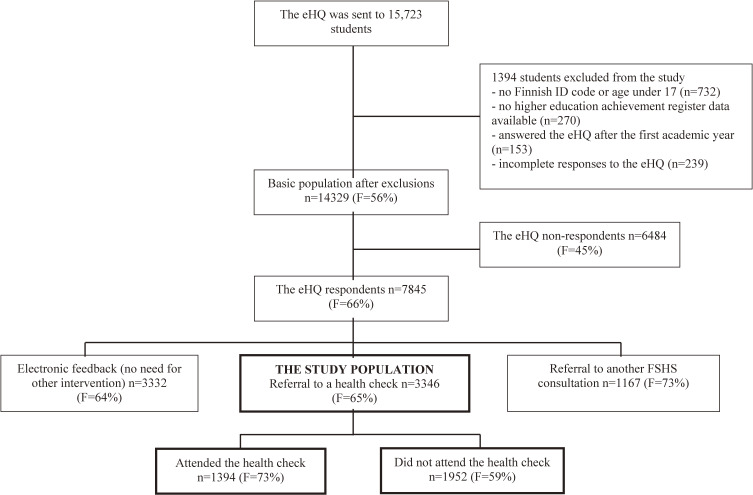
Proportions of females (F) presented in parentheses for each step of the health examination process.

This was a nationwide register-based cohort study in Finland, the design and methodology of which have been described previously [10]. The study was conducted using data on the health of university entrants produced during the FSHS health examination process. When admitted to a university, students are granted the right to study undergraduate studies followed by graduate studies. The word entrants in this study signifies students admitted to undergraduate studies. The basic population was the national cohort of university entrants from the 2011–2012 academic year in Finland (n = 15,723) who were followed for six-years. The final study population in the present study consisted of the 3346 students who were referred to a health check based on their eHQ responses (Fig. 1). The students who attended and did not attend the health check were compared.

### The electronic health questionnaire (eHQ)

The eHQ was developed and piloted in 2009 for practical purposes of FSHS [11]. The questionnaire comprised 26 questions about health, social relations, and studying (Additional file 1). The questionnaire was conducted in the two official languages (Finnish and Swedish), and additionally in English. The students received an invitation to respond to the eHQ by email. The email included a link to log in to the web-based eHQ program where they could respond to the questionnaire. The program sent an automated remainder once, two weeks after the initial invitation had been sent. The responses were read and considered by the FSHS public health nurses. Based on the eHQ responses, a nurse guides the student to one of the following interventions: 1) referral to a health check conducted by a public health nurse, 2) referral to an appointment other than a health check, e.g., physiotherapy, or 3) in the case of no need for other interventions, an electronic message to the student to support a healthy lifestyle. The public health nurse gave the feedback in the eHQ program, and the student received an email notification to log in to the eHQ program to check their feedback.

Ten of the eHQ questions had a response scale from −10 to +10. Respondents were guided to interpret the scale so that positive numbers suggested a favourable status, and zero (0) suggested a neutral situation, and negative numbers suggested a problematic situation. As the distributions of the responses were highly skewed to the high positive end of the scale, we wanted to examine whether also low positive values indicated a problematic situation. The responses were therefore sorted into three categories for statistical analysis as High (8–10), Medium (0–7) and Low (−10–−1).

Drug use was assessed by asking: “Have you experimented or used any drugs or taken alcohol and medication at the same time in order to get intoxicated?” The response alternatives were: “never, yes 1–4 times, yes 5 times or more often”. For the statistical analysis, the latter two responses were combined into one “yes” category.

Alcohol use was assessed by asking: “Do you use alcohol?”. Subsequent to the response “yes”, the 10-item Alcohol Use Disorders Identification Test (AUDIT) was presented [13]. AUDIT points were calculated and categorised into four categories according to the World Health Organization (WHO) classification: <8 low risk, 8–15 medium risk, 16–20 high risk and >20 possible alcohol dependence [14].

Age was categorised as in the Eurostudent study and in the Finnish University Students’ Health Survey as follows: 17–21 years, 22–24 years, 25–29 years and 30 years or older [15, 16]. Students reported their height and weight in the eHQ. Body-mass index (BMI) was calculated and categorised following the WHO categorisation: <18.5 (underweight), 18.5–24.99 (normal weight), 25.0–29.99 (overweight), 30–34.99 (obese, Class I), and ≥35 (obese, Class II and III) [17].

The eHQ data included register-based information about the students’ faculty of study. For the statistical analyses the faculties were categorised to form the variable “field of study”. The categorisation was based on the classification used in the Finnish University Students’ Health Survey and is in accordance with the fields of study listed by the Ministry of Education and Culture. Faculties were categorised as “other” when they could be included in more than one category.

### Data

The eHQ data were obtained from the eHQ register of the FSHS for the 2011–2012 academic year. The intervention chosen by the public health nurse was available from the eHQ data. The information about students’ attendance of the health check was collected from the FSHS medical records. The data were linked by using Finnish personal identity codes [18]. All Finnish citizens and permanent residents have personal identity codes administered by the Digital and Population Data Services Agency, which enables data linkage between the registers and individual-level analyses. The information about the sex of the students was based on the personal identity codes.

### Statistical analysis

To describe the data, the frequencies and percentages of each variable were calculated. Chi-squared tests were employed to detect associations between the categorical variables. In cases with continuous non-normally distributed data, the Mann–Whitney-*U*-test was used to detect the differences between groups. A p-value <0.05 was considered to be statistically significant.

The data were analysed with a binary logistic regression model to detect the variables in the eHQ that were statistically significantly associated with health check non-attendance. In the logistic regression models, the odds ratios of the categorical variables were compared against the reference category of each variable, with the exception that the field of study was compared against the mean of all study fields. First, univariate models were created for each associated factor separately. Further, as many of the students may have several risk factors, we used multivariable logistic regression, into which we included all the variables with p < 0.05 between the outcome and independent variable. With this model we computed the odds ratios (OR) with 95% confidence intervals (CI), now adjusted for other covariates, including possible confounders. All statistical analyses were carried out using IBM SPSS Statistics for Windows, version 26 (IBM Corp) and R version 3.6.1 (The R Foundation), with package ggplot2.

## Results

Of the university entrants who responded to the eHQ (n = 7845) 43% were referred to a health check (n = 3346) of which at total 58% did not attend (Fig. 1). Table 1 shows the demographics of the students referred to the health check by the health check attendance. Of the students referred to the health check, 68% of men and 53% of women did not attend (p < 0.001) (Table 1). There was no difference in age groups between the students who attended and did not attend the health check (p = 0.056). The proportion of the non-attendees was highest among law students (66%) and students categorized into the “other” group (74%) and it was the lowest among arts students (42%).

**Table 1:** Demographics of the university entrants referred to a health check (n = 3346) by health check attendance.

	**Total** **(n = 3346)**		**Did not attend the health check** **(n = 1952)**		**Attended the health check** **(n = 1388)**		**p-value**
	**%**	**n**	**%**	**n**	**%**	**n**	
**Sex**							<0.001
Female	63	2163	53	1152	47	1011	
Male	35	1183	68	800	32	383	
**Age**							0.056
17–21	64	2170	60	1299	40	871	
22–24	14	461	57	261	43	200	
25–29	12	422	53	224	47	198	
≥30	9	293	57	168	43	125	
**Field of study**							<0.001
Law	3	89	66	58	34	31	
Business and economics	8	224	64	160	36	64	
Technology and engineering	21	787	63	468	37	319	
Medicine	4	119	59	68	41	51	
Natural sciences, agriculture and forestry, and pharmacy	17	515	57	301	43	214	
Social sciences	15	362	57	204	43	158	
Sports science, educational sciences, health sciences, psychology	14	439	55	241	45	198	
Humanities, theology, philosophy	17	564	54	309	46	255	
Arts	3	152	42	74	58	78	
Other	3	95	74	69	26	26	

P-values result from the Chi-square tests and describe differences in sex, age and field of study between students who attended and did not attend the health check.

The Table 2 presents the distributions of the eHQ responses, in which there was a statistical difference between the health check non-attendees and attendees. Further, all the distributions are presented in Additional file 2. Of the students who did not attend the health check, 43% got eight (8) points or more in the AUDIT, referring to possible risks of alcohol use. This was statistically higher proportion (p < 0.001) compared to the 34% of the attendees. The non-attendees were more often daily or occasional smokers (31%) than the attendees (24%) (p < 0.001). Further, there were statistical differences in the frequency of reported chronic diseases (p = 0.012) and recurrent symptoms (p < 0.001) of which non-attendees reported 24% and 41% while the percentages among attendees were 27% and 52%, respectively. Additionally, of the non-attendees, 11% reported low general health and 12% low usual state of mind, while the percentages for the attendees were statistically higher, 14% and 17%, respectively.

**Table 2:** The eHQ responses of university entrants referred to a health check by health check attendance.

**The eHQ responses**	**Did not attend the health check** **(n = 1952)**		**Attended the health check** **(n = 1394)**		**p-value**
	**%**	**n**	**%**	**n**	
**Studying**					
Enthusiasm about the field of study on a −10 to +10 scale					0.046
high 8–10	49	953	52	722	
medium 0–7	41	805	41	565	
low −10–−1	10	194	8	107	
Engagement with studies on a −10 to +10 scale					0.006
high 8–10	25	496	30	422	
medium 0–7	60	1167	57	792	
low −10–−1	15	289	13	180	

**Health habits**					
Alcohol use					<0.001
do not use	16	313	19	268	
AUDIT 1–7 points	40	782	47	657	
AUDIT 8–15 points	36	711	28	389	
AUDIT 16–19 points	4	87	4	54	
AUDIT ≥ 20 points	3	59	2	26	
Smoking ore use of other tobacco products					<0.001
no	68	1330	75	1046	
occasionally	20	400	16	230	
daily	11	222	8	118	

**General health**					
Reported chronic diseases					0.012
no	76	1492	73	1012	
yes	24	460	27	382	
Reported persistent or recurrent symptoms					<0.001
no	59	1145	48	668	
yes	41	807	52	726	
General health status on a −10 to +10 scale					0.001
high 8–10	35	689	30	424	
medium 0–7	54	1054	56	775	
low −10–−1	11	209	14	195	

**Dental health**					
Teeth brushing					0.018
twice a day or more often	67	1307	71	988	
once a day	31	598	28	386	
less than once a day	2	47	1	20	

**Mental well-being and social relations**					
Normal attitude towards food					0.004
yes	75	1472	70	980	
no	7	141	8	118	
can not say	17	339	21	296	
Usual state of mind on a −10 to +10 scale					0.001
high 8–10	27	520	23	327	
medium 0–7	61	1189	60	832	
low −10–−1	12	243	17	235	

**Other issues**					
Indicates willingness to discuss about sexual health					<0.001
no	87	1689	78	1085	
yes	13	263	22	309	
Indicates willingness to discuss about a non-specific matter					<0.001
no	78	1518	62	869	
yes	22	434	38	525	

Number of university entrants referred to the health check n = 3346. Differences between non-attendees and attendees were tested with the Chi-Square test. The statistically significant differences in distributions are presented.

Table 3 shows unadjusted and adjusted ORs for variables present in the final model of the multivariable logistic regression analysis which describes the associations between the students eHQ responses and the health check non-attendance. Unadjusted and adjusted ORs for all variables in the binary logistic regression models are presented in Additional file 3. The variable with the highest OR for health check non-attendance was the male sex, as males were 1.59 times more likely not to attend the health check when compared to women (OR = 1.59, 95% CI 1.35–1.87) (Table 3). Further, low engagement with studies (OR = 1.54, 95% CI 1.20–1.99) and both daily (OR = 1.30, 95% CI 1.01–1.67) and occasional (OR = 1.30, 95% CI 1.08–1.57) smoking were associated with health check non-attendance. Business and economics (OR = 1.50, 95% CI (1.12–1.99)) and the group other (OR = 1.57, 95% CI (1.03–2.41)) were the fields of study with the highest OR for non-attendance. Conversely, indicated willingness to discuss about sexual health (OR = 0.67, 95% CI (0.56–0.81)) or other matters (OR = 0.55, 95% CI (0.47–0.64)), low usual state of mind (OR = 0.64, 95% CI (0.50–0.82)), and the presence of persistent or recurrent symptoms (OR = 0.77, 95% CI (0.67–0.89)) were negatively associated with health check non-attendance.

**Table 3:** Univariate and multivariable logistic regression analysis showing predictors of health check non-attendance.

	**Unadjusted OR** **(95% CI)**	**Adjusted OR** **(95% CI)**
**Demographics**		
**Sex**		
Female	1.00	1.00
Male	1.83 (1.58–2.13)	1.59 (1.35–1.87)
**Field of study**		
Humanities, theology, philosophy	0.80 (0.67–0.96)	0.94 (0.78–1.13)
Social sciences	0.86 (0.70–1.05)	0.87 (0.70–1.08)
Law	1.24 (0.83–1.85)	1.20 (0.79–1.81)
Natural sciences, agriculture, and forestry, pharmacy	0.93 (0.78–1.12)	0.89 (0.74–1.08)
Business and economics	1.66 (1.26–2.18)	1.50 (1.12–1.99)
Technology and engineering	0.97 (0.83–1.14)	0.79 (0.67–0.94)
Other	1.76 (1.16–2.66)	1.57 (1.03–2.41)
Sports science, educational sciences, health sciences, psychology	0.81 (0.67–0.98)	0.95 (0.78–1.17)
Arts	0.63 (0.47–0.85)	0.71 (0.52–0.96)
Medicine	0.88 (0.63–1.24)	0.91 (0.64–1.29)

**The eHQ responses**		
**Studying**		
Engagement to studies on scale −10–+10		
high 8–10	1.00	1.00
medium 0–7	1.25 (1.07–1.47)	1.26 (1.06–1.50)
low −1–−10	1.37 (1.09–1.71)	1.54 (1.20–1.99)

**Health habits**		
Smoking		
no	1.00	
occasionally	1.37 (1.14–1.645)	1.30 (1.08–1.57)
daily	1.48 (1.17–1.88)	1.30 (1.01–1.67)

**General health**		
Persistent or recurrent symptoms		
no	1.00	1.00
yes	0.65 (0.57–0.75)	0.77 (0.67–0.89)

**Mental well-being and social relations**		
Usual state of mind on scale −10–+10		
high 8–10	1.00	1.00
medium 0–7	0.90 (0.76–1.06)	0.84 (0.70–1.00)
low −1–−10	0.65 (0.52–0.82)	0.64 (0.50–0.82)

**Other issues**		
Indicates willingness to discuss about sexual health		
no	1.00	1.00
yes	0.55 (0.46–0.66)	0.67 (0.56–0.82)
Indicates willingness to discuss about a non-specific matter		
no	1.00	1.00
yes	0.47 (0.41–0.55)	0.55 (0.47–0.64)

Unadjusted and adjusted odd ratios (OR) with 95% confidential interval (CI). The reference of the OR for field of study is the mean of all study fields.

## Discussion

In this study, low engagement with studies, smoking and male sex were associated with health check non-attendance. Low usual state of mind, the presence of persistent or recurrent symptoms and indicated willingness to discuss with a health care professional were negatively associated with health check non-attendance, thus enhanced the health check attendance in student health care.

This study found that university entrants’ low engagement with studies was associated with non-attendance of the health check in student health care, a novel finding. It is possible that entrants with low engagement with studies did not find the health check respond to their needs. In the Finnish University Students’ Health Survey students have repeatedly reported a need for help to deal with stress and time management [15], factors influencing study engagement [19, 20]. As these issues have been responsibility of student counselling, and less of student health care, there seems to be a need for improving collaboration.

The present results support previous findings about the association between smoking and non-attendance of health checks [4, 5]. In the university student population, smoking has been associated with several risk factors like binge drinking, drug consumption, and low belief ratings in the health benefits of not smoking [21, 22]. Adolescence is a transitional phase in which the opportunities for health promotion can be great, highlighting the importance of motivating young adult smokers to attend the health checks.

Support for previous findings on male sex associating with non-attendance of health checks is presented in this study [23–25]. In a review, male-dominant barriers to attend health screening included heterosexual self-presentation, avoidance of femininity and lack of time [24]. Another systematic review stated that there is little published evidence on how to improve men’s uptake of health promotion services [25].

The present results indicate that having recurrent symptoms or low usual state of mind are negatively associated with health check non-attendance in university student population. These findings are contradictory to previous research conducted in older adult populations, where non-attenders in routine health checks appeared to have greater clinical needs [4, 26]. It is possible that university students’ help seeking behaviour differs from other adult populations.

Poor mental health of university students and young people in general has been a growing public concern [27–29]. It was an encouraging finding that students with low usual state of mind were 1,6 times more likely to attend the health check than those with a good state of mind. This is supported by one previous research which has shown that students who participate in the first stage of the health examination process of FSHS, i.e. respond to the eHQ, have more health problems, especially mental health issues, than non-participants [30]. However, in most previous studies, psychological issues of college students have been associated with restrains in seeking medical services [31–33]. It should be considered that the health examination process of the FSHS may provide the students a low threshold gateway to mental health services.

In the eHQ, the students were able to indicate their willingness to discuss with a health care professional which according to the present results was negatively associated with health check non-attendance. It is possible that these students had health concerns and suitable timing of the eHQ offered them an easy access to health services, explaining the finding. Previously described facilitators of health check attendance as feeling responsible for one’s health, finding health important and believing to be able to influence one’s own health may explain the finding as well [34].

Despite the large number of variables examined, health- or health habit-related reasons did not seem to be associated with the health check non-attendance to a great extent in the university student population. In previous studies, conducted in non-student populations, the reasons for not attending general health checks have often been non-health-related, including lack of awareness, long distances or other difficulties with access to the health care, and time constraints [4, 8, 9]. It has been stated that young people are often unwilling or unable to obtain needed health services, indicating barriers related to the availability, accessibility and acceptability of health services [35]. We expect factors such as these to partially explain the health check non-attendance of university students, however they were not in a scope of this study and should be a subject for further research.

### Strengths and limitations

The greatest strengths of this study were the wide variety of factors studied, the real-life setting and using register data on a national cohort of university entrants. This was the first study to address the reasons for health check non-attendance in a student health care setting. The reasons for the lack of previous research can be diverse. In general, health checks are understudied [2] which could be due to the strong tradition they have in preventive health care. Further, the global concern about students’ mental health [28] might have directed the research resources to psychosocial factors.

There are inherent limitations in the health examination process of the FSHS, and the real-life design of this study. The eHQ was developed for practical purposes and was validated accordingly, and not to the degree of scientific rigor [10]. The eHQ data are self-reported data and are therefore susceptible to bias [36]. As the response rate to the eHQ was fairly low (55%), almost half of university entrants did not have their needs for a health check screened.

## Conclusions

The results suggest that providing health checks may serve as a way of reaching students with health concerns, which could translate into opportunities to incur health benefits of general health checks in student health care. However, motivating males and smokers to attend general health checks continue to be a challenge also in a university student population. That low engagement with studies associates with health check non-attendance points to clear need to improve collaboration between universities and student health care.

## Abbreviations

eHQ: electronic health questionnaire
FSHS: the Finnish Student Health Service
AUDIT: Alcohol Use Disorders Identification Test
BMI: body mass index
WHO: World Health Organization

## Supplementary information

**Additional file 1:** The electronic health questionnaire of the Finnish Student Health Service.

**Additional file 2:** The eHQ responses of the university entrants referred to a health check (n = 3,346) by their health check attendance. Differences between non-attendees and attendees were tested with the Chi-Square test.

**Additional file 3:** Association between eHQ responses and health check non-attendance modelled by binary logistic regression analysis. Unadjusted and adjusted odd ratios (OR) with 95% confidence intervals (CI) presented.

## Declarations

### Availability of data and materials

The data that support the findings of this study are available from the Finnish Student Health Service, but restrictions apply to the availability of these data. In this study, the data were used under license granted by the Finnish Institute for Health and Welfare (Dnro THL/1364/5.05.00/2017) which forbids data sharing, and therefore the data are not publicly available.

## References

[1] Si S, Moss JR, Sullivan TR, . Effectiveness of general practice-based health checks: a systematic review and meta-analysis. Br J Gen Pract. 2014;64:e47–53. 24567582 10.3399/bjgp14X676456 PMC3876170

[2] Krogsbøll LT, Jørgensen KJ, Gøtzsche PC. General health checks in adults for reducing morbidity and mortality from disease. Cochrane Database Syst Rev. 2019. doi: 10.1002/14651858.CD009009.pub3. PMC6353639 30699470

[3] Martin A, Saunders CL, Harte E, . Delivery and impact of the NHS Health Check in the first 8 years: A systematic review. Br J Gen Pract. 2018;68:e449–59. 29914882 10.3399/bjgp18X697649 PMC6014431

[4] Dryden R, Williams B, McCowan C, . What do we know about who does and does not attend general health checks? Findings from a narrative scoping review. BMC Public Health. 2012;12:723. 22938046 10.1186/1471-2458-12-723 PMC3491052

[5] Hoebel J, Starker A, Jordan S, . Determinants of health check attendance in adults: Findings from the cross-sectional German Health Update (GEDA) study. BMC Public Health. 2014;14:913. 25185681 10.1186/1471-2458-14-913 PMC4167266

[6] Brunner-Ziegler S, Rieder A, Stein KV, . Predictors of participation in preventive health examinations in Austria. BMC Public Health. 2013;13:1138. 24308610 10.1186/1471-2458-13-1138 PMC3866300

[7] Thorogood M, Coulter A, Jones L, . Factors affecting response to an invitation to attend for a health check. J Epidemiol Community Health. 1993;47:224–8. 8350036 10.1136/jech.47.3.224 PMC1059771

[8] Harte E, MacLure C, Martin A, . Reasons why people do not attend NHS Health Checks: a systematic review and qualitative synthesis. Br J Gen Pract. 2018;68:e28–35. 29203682 10.3399/bjgp17X693929 PMC5737317

[9] Tolonen H, Lundqvist A, Jääskeläinen T, . Reasons for non-participation and ways to enhance participation in health examination surveys - The Health 2011 Survey. Eur J Public Health. 2017;27:909–11. 28957480 10.1093/eurpub/ckx098

[10] Paldanius S, Seilo N, Kunttu K, . Screening University Students for Health Checks With an Electronic Health Questionnaire in Finland: Protocol for a Retrospective, Register-Based Cohort Study. JMIR Res Protoc. 2020;9:e14535. 32012093 10.2196/14535 PMC7016620

[11] Kunttu K, Huttunen T. Advance screening in two-stage health examination among first-year university students [Lyhyt terveyskysely tunnistaa uuden opiskelijan terveysriskit] (summary in English). Finnish Med J. 2008;63:3216–22.

[12] Health Care Act (1326/2010). 30.12.2010/1326.

[13] Reinert DF, Allen JP. The alcohol use disorders identification test: An update of research findings. Alcohol Clin Exp Res. 2007;31:185–99. 17250609 10.1111/j.1530-0277.2006.00295.x

[14] Babor TF, Higgins-Biddle JC, Saunders JB, et al. The Alcohol Use Disorders Identification Test Guidelines for Use in Primary Care. 2001.

[15] Kunttu K, Pesonen T, Saari J. Student Health Survey 2016: a national survey among Finnish university students. Helsinki, https://www.yths.fi/en/fshs/research-and-publications/the-finnish-student-health-survey-2/ (accessed 12 April 2022).

[16] Hauschildt K, Vögtle EM, Gwosc C. *EUROSTUDENT VI* Overview and selected findings. Social and Economic Conditions of Student Life in Europe. Bielefeld. 2018. doi: 10.3278/104-274w.

[17] Obesity: preventing and managing the global epidemic. Report of a WHO Consultation (WHO Technical Report Series 894). Geneva, 2000. 11234459

[18] Digital and Population Data Services Agency - The personal identity code, https://dvv.fi/en/personal-identity-code (accessed 12 April 2022).

[19] Liu H, Yansane AI, Zhang Y, . Burnout and study engagement among medical students at Sun Yat-sen University, China: A cross-sectional study. Medicine. 2018;97(15):e0326. doi: 10.1097/MD.0000000000010326. 29642167 PMC5908607

[20] Bilge F, Tuzgöl Dost M, Çetin B. Factors affecting burnout and school engagement among high school students: Study habits, self-efficacy beliefs, and academic success. Kuram ve Uygulamada Egit Bilim. 2014;14:1721–7.

[21] Steptoe A, Wardle J, Cui W, . An international comparison of tobacco smoking, beliefs and risk awareness in university students from 23 countries. Addiction. 2002;97:1561–71. 12472640 10.1046/j.1360-0443.2002.00269.x

[22] El Ansari W, Stock C. Factors associated with smoking, quit attempts and attitudes towards total smoking bans at university: a survey of seven universities in England, Wales and Northern Ireland. Asian Pac J Cancer Prev. 2012;13:705–14. 22524848 10.7314/apjcp.2012.13.2.705

[23] Bunten A, Porter L, Gold N, . A systematic review of factors influencing nhs health check uptake: Invitation methods, patient characteristics, and the impact of interventions. BMC Public Health. 2020;20:93. 31964366 10.1186/s12889-019-7889-4 PMC6975079

[24] Teo CH, Ng CJ, Booth A, . Barriers and facilitators to health screening in men: A systematic review. Soc Sci Med. 2016;165:168–76. 27511617 10.1016/j.socscimed.2016.07.023

[25] Robertson LM, Douglas F, Ludbrook A, . What works with men? A systematic review of health promoting interventions targeting men. BMC Health Serv Res. 2008;8:141. 18598339 10.1186/1472-6963-8-141 PMC2483970

[26] Culica D, Rohrer J, Ward M, . Medical checkups: Who does not get them? Am J Public Health. 2002;92:88–91. 11772768 10.2105/ajph.92.1.88 PMC1447395

[27] Patel V, Flisher AJ, Hetrick S, . Mental health of young people: a global public-health challenge. Lancet. 2007;369:1302–13. 17434406 10.1016/S0140-6736(07)60368-7

[28] Auerbach RP, Alonso J, Axinn WG, . Mental disorders among college students in the World Health Organization World Mental Health Surveys. Psychol Med. 2016;46:2955–70. 27484622 10.1017/S0033291716001665 PMC5129654

[29] Donato F, Triassi M, Loperto I, . Symptoms of mental health problems among Italian adolescents in 2017–2018 school year: a multicenter cross-sectional study. Environ Health Prev Med. 2021;26(1):67. doi: 10.1186/s12199-021-00988-4. 34154531 PMC8216089

[30] Ritakorpi M, Kaunonen M, Kaila M, . The non-response of university students to an electronic health questionnaire - non-response analysis. [Sähköiseen terveyskyselyyn vastaamatta jättäneet yliopisto-opiskelijat]. (Summary in English). J Soc Med. 2019;56:1. doi: 10.23990/sa.70440.

[31] Tran DMT, Silvestri-Elmore A. Healthcare-seeking behaviours in college students and young adults: a review. J Res Nurs. 2021;26(4):320–38. doi: 10.1177/1744987120951594. 35251258 PMC8894990

[32] Hunt J, Eisenberg D. Mental Health Problems and Help-Seeking Behavior Among College Students. J Adolesc Health. 2010;46:3–10. 20123251 10.1016/j.jadohealth.2009.08.008

[33] Vidourek RA, King KA, Nabors LA, . Students’ benefits and barriers to mental health help-seeking. Health Psychol Behav Med. 2014;2:1009–22. 25750831 10.1080/21642850.2014.963586 PMC4346065

[34] de Waard AKM, Wändell PE, Holzmann MJ, . Barriers and facilitators to participation in a health check for cardiometabolic diseases in primary care: A systematic review. Eur J Prev Cardiol. 2018;25:1326–40. 29916723 10.1177/2047487318780751 PMC6097107

[35] Tylee A, Haller DM, Graham T, . Youth-friendly primary-care services: how are we doing and what more needs to be done? Lancet. 2007;369:1565–73. 17482988 10.1016/S0140-6736(07)60371-7

[36] Brener ND, Billy JOG, Grady WR. Assessment of factors affecting the validity of self-reported health-risk behavior among adolescents: Evidence from the scientific literature. J Adolesc Health. 2003;33:436–57. 14642706 10.1016/s1054-139x(03)00052-1

