# Supplementary material for: Health check attendance association with health and study-related factors: a register-based cohort study of Finnish university entrants
Source: Environ Health Prev Med. 2022 Aug 19;27:34. doi: 10.1265/ehpm.22-00032 (PMC9425058; doi:10.1265/ehpm.22-00032)
Supplement: Supplementary file 2 — Additional file 2: The eHQ responses of the university entrants referred to a health check (n = 3,346) by their health check attendance. Differences between non-attendees and attendees were tested with the Chi-Square test. [file ehpm-27-034-s002.docx]

| **The eHQ responses** | **Did not attend the health check (n=1952)** | | **Attended the health check (n=1394)** | | **p-value** |
| --- | --- | --- | --- | --- | --- |
|  | % | n | % | n |  |
| **Studying** |  |  |  |  |  |
| Enthusiasm about the field of study on a -10 – +10 scale | |  |  |  | 0.046 |
| high 8–10 | 49 | 953 | 52 | 722 |  |
| medium 0–7 | 41 | 805 | 41 | 565 |  |
| low -10 – -1 | 10 | 194 | 8 | 107 |  |
| Engagement to studies on a -10 – +10 scale |  |  |  |  | 0.006 |
| high 8–10 | 25 | 496 | 30 | 422 |  |
| medium 0–7 | 60 | 1167 | 57 | 792 |  |
| low -10 – -1 | 15 | 289 | 13 | 180 |  |
|  |  |  |  |  |  |
| **Health habits** |  |  |  |  |  |
| Exercise |  |  |  |  | 0.951 |
| yes | 79 | 1533 | 79 | 1096 |  |
| no | 21 | 419 | 21 | 298 |  |
| Alcohol use |  |  |  |  | <0.001 |
| do not use | 16 | 313 | 19 | 268 |  |
| AUDIT 1–7 points | 40 | 782 | 47 | 657 |  |
| AUDIT 8–15 points | 36 | 711 | 28 | 389 |  |
| AUDIT 16–19 points | 4 | 87 | 4 | 54 |  |
| AUDIT >= 20 points | 3 | 59 | 2 | 26 |  |
| Used/tried drugs |  |  |  |  | 0.148 |
| no | 80 | 1563 | 82 | 1144 |  |
| yes | 20 | 389 | 18 | 250 |  |
| Smoking ore use of other tobacco products |  |  |  |  | <0.001 |
| no | 68 | 1330 | 75 | 1046 |  |
| occasionally | 20 | 400 | 16 | 230 |  |
| daily | 11 | 222 | 8 | 118 |  |
| Healthiness of eating habits on a -10 – +10 scale |  |  |  |  | 0.809 |
| high 8–10 | 16 | 317 | 16 | 219 |  |
| medium 0–7 | 65 | 1260 | 66 | 915 |  |
| low -10 – -1 | 19 | 375 | 19 | 260 |  |
| Adequacy and quality of sleeping on a -10 – +10 scale |  |  |  |  | 0.581 |
| high 8–10 | 18 | 353 | 19 | 266 |  |
| medium 0–7 | 48 | 930 | 46 | 640 |  |
| low -10 – -1 | 34 | 669 | 35 | 488 |  |
| Leisure time in terms of recovery, recreation and relaxation on a -10 – +10 scale |  |  |  |  | 0.554 |
| high 8–10 | 40 | 789 | 39 | 548 |  |
| medium 0–7 | 52 | 1015 | 52 | 727 |  |
| low -10 – -1 | 8 | 148 | 9 | 119 |  |
|  |  |  |  |  |  |
| **General health** |  |  |  |  |  |
| Reported chronic diseases |  |  |  |  | 0.012 |
| no | 76 | 1492 | 73 | 1012 |  |
| yes | 24 | 460 | 27 | 382 |  |
| Reported persistent or recurrent symptoms |  |  |  |  | <0.001 |
| no | 59 | 1145 | 48 | 668 |  |
| yes | 41 | 807 | 52 | 726 |  |
| General health status on a -10 – +10 scale |  |  |  |  | 0.001 |
| high 8–10 | 35 | 689 | 30 | 424 |  |
| medium 0–7 | 54 | 1054 | 56 | 775 |  |
| low -10 – -1 | 11 | 209 | 14 | 195 |  |
| Body mass index |  |  |  |  | 0.277 |
| < 18,5 (underweight) | 8 | 148 | 9 | 130 |  |
| 18.5–24.99 (normal weight) | 65 | 1269 | 65 | 901 |  |
| 25–29.99 (overweight) | 18 | 353 | 18 | 253 |  |
| 30–34.99 (obese class I) | 6 | 122 | 6 | 79 |  |
| ≥35 (obese class II and III) | 2 | 48 | 2 | 25 |  |
|  |  |  |  |  |  |
| **Dental health** |  |  |  |  |  |
| Latest dental check-up |  |  |  |  | 0.402 |
| 0–2 years ago | 62 | 1212 | 62 | 861 |  |
| 3–5 years ago | 31 | 608 | 32 | 453 |  |
| more than 5 years ago | 7 | 132 | 6 | 80 |  |
| Eating and drinking times per day |  |  |  |  | 0.682 |
| 6 or less | 79 | 1545 | 80 | 1118 |  |
| 7–10 | 19 | 372 | 18 | 255 |  |
| more than 10 | 2 | 35 | 2 | 21 |  |
| Teeth brushing |  |  |  |  | 0.018 |
| twice a day or more often | 67 | 1307 | 71 | 988 |  |
| once a day | 31 | 598 | 28 | 386 |  |
| less than once a day | 2 | 47 | 1 | 20 |  |
| Cavities that require filling at dental check–ups |  |  |  |  | 0.258 |
| never | 25 | 490 | 23 | 318 |  |
| seldom | 48 | 944 | 49 | 682 |  |
| often or every time | 27 | 518 | 28 | 394 |  |
| Dental fear |  |  |  |  | 0.084 |
| not at all | 60 | 1167 | 56 | 780 |  |
| some | 34 | 656 | 37 | 516 |  |
| very much | 7 | 129 | 7 | 98 |  |
|  |  |  |  |  |  |
| **Mental well–being and social relations** |  |  |  |  |  |
| Normal attitude towards food |  |  |  |  | 0.004 |
| yes | 75 | 1472 | 70 | 980 |  |
| no | 7 | 141 | 8 | 118 |  |
| can't say | 17 | 339 | 21 | 296 |  |
| Usual state of mind on a -10 – +10 scale |  |  |  |  | 0.001 |
| high 8–10 | 27 | 520 | 23 | 327 |  |
| medium 0–7 | 61 | 1189 | 60 | 832 |  |
| low -10 – -1 | 12 | 243 | 17 | 235 |  |
| Experience of loneliness on a -10 – +10 scale |  |  |  |  | 0.322 |
| high 8–10 | 34 | 660 | 36 | 497 |  |
| medium 0–7 | 43 | 840 | 40 | 564 |  |
| low -10 – -1 | 23 | 452 | 24 | 333 |  |
| Relationship with the parents on a -10 – +10 scale |  |  |  |  | 0.031 |
| high 8–10 | 53 | 1028 | 53 | 739 |  |
| medium 0–7 | 41 | 807 | 39 | 541 |  |
| low -10 – -1 | 6 | 117 | 8 | 114 |  |
| Experiencing various social situations (etc. giving presentation) on a -10 – +10 scale |  |  |  |  | 0.119 |
| high 8–10 | 21 | 416 | 19 | 260 |  |
| medium 0–7 | 46 | 901 | 46 | 647 |  |
| low -10 – -1 | 33 | 635 | 35 | 487 |  |
|  |  |  |  |  |  |
| **Other issues** |  |  |  |  |  |
| Presents willingness to discuss about sexual health |  |  |  |  | <0.001 |
| no | 87 | 1689 | 78 | 1085 |  |
| yes | 13 | 263 | 22 | 309 |  |
| Presents willingness to discuss about a non–specific matter |  |  |  |  | <0.001 |
| no | 78 | 1518 | 62 | 869 |  |
| yes | 22 | 434 | 38 | 525 |  |
