# Supplementary material for: Health check attendance association with health and study-related factors: a register-based cohort study of Finnish university entrants
Source: Environ Health Prev Med. 2022 Aug 19;27:34. doi: 10.1265/ehpm.22-00032 (PMC9425058; doi:10.1265/ehpm.22-00032)
Supplement: Supplementary file 3 — Additional file 3: Association between eHQ responses and health check non-attendance modelled by binary logistic regression analysis. Unadjusted and adjusted odd ratios (OR) with 95% confidence intervals (CI) presented. [file ehpm-27-034-s003.docx]

|  | Unadjusted OR (95% CI) | Adjusted OR (95% CI) |
| --- | --- | --- |
| **Demographics** |  |  |
| Sex |  |  |
| Female | 1.00 | 1.00 |
| Male | 1.83 (1.58-2.13) | 1.59 (1.35-1.87) |
| Age at enrolment |  |  |
| 17–21 | 1.00 |  |
| 22–24 | 0.88 (0.71-1.07) |  |
| 25–29 | 0.76 (0.62-0.94) |  |
| 30 or older | 0.90 (0.70-1.15) |  |
| Field of study |  |  |
| Humanities, theology, philosophy | 0.80 (0.67-0.96) | 0.94 (0.78-1.13) |
| Social sciences | 0.86 (0.70-1.05) | 0.87 (0.70-1.08) |
| Law | 1.24 (0.83-1.85) | 1.20 (0.79-1.81) |
| Natural sciences, agriculture and forestry,  pharmacy | 0.93 (0.78-1.12) | 0.89 (0.74-1.08) |
| Business and economics | 1.66 (1.26-2.18) | 1.50 (1.12-1.99) |
| Technology and engineering | 0.97 (0.83-1.14) | 0.79 (0.67-0.94) |
| Other | 1.76 (1.16-2.66) | 1.57 (1.03-2.41) |
| Sports science, educational sciences,  health sciences, psychology | 0.81 (0.67-0.98) | 0.95 (0.78-1.17) |
| Arts | 0.63 (0.47-0.85) | 0.71 (0.52-0.96) |
| Medicine | 0.88 (0.63-1.24) | 0.91 (0.64-1.29) |
|  |  |  |
| **Studying** |  |  |
| Enthusiasm about studying on scale -10 - +10 |  |  |
| high 8–10 | 1.00 |  |
| medium 0–7 | 1.08 (0.93-1.25) |  |
| low -1 – -10 | 1.37 (1.06-1.77) |  |
| Engagement to studies on scale -10 – +10 |  |  |
| high 8–10 | 1.00 | 1.00 |
| medium 0–7 | 1.25 (1.07-1.47) | 1.26 (1.06-1.50) |
| low -1 – -10 | 1.37 (1.09-1.71) | 1.54 (1.20-1.99) |
|  |  |  |
| **Health habits** |  |  |
| Alcohol use |  |  |
| Don't use alcohol | 1.00 |  |
| AUDIT 1–7 points | 1.02 (0.84-1.24) |  |
| AUDIT 8–15 points | 1.57 (1.28-1.92) |  |
| AUDIT 16–19 points | 1.38 (0.95-2.01) |  |
| AUDIT ≥20 points | 1.94 (1.19-3.17) |  |
| Smoking |  |  |
| no | 1.00 |  |
| occasionally | 1.37 (1.14-1.645) | 1.30 (1.08-1.57) |
| daily | 1.48 (1.17-1.88) | 1.30 (1.01-1.67) |
|  |  |  |
| **General health** |  |  |
| Chronic diseases |  |  |
| no | 1.00 |  |
| yes | 0.82 (0.70-0.96) |  |
| Persistent or recurrent symptoms |  |  |
| no | 1.00 | 1.00 |
| yes | 0.65 (0.57-0.75) | 0.77 (0.67-0.89) |
| General health status on scale -10 – +10 |  |  |
| high 8–10 | 1.00 |  |
| medium 0–7 | 0.84 (0.72-0.98) |  |
| low -1 – -10 | 0.66 (0.52-0.83) |  |
|  |  |  |
| **Dental health** |  |  |
| Teeth brushing |  |  |
| twice a day or more often | 1.00 |  |
| once a day | 1.17 (1.01-1.36) |  |
| less than once a day | 1.78 (1.05-3.02) |  |
| Dental fear |  |  |
| not at all | 1.00 |  |
| some | 0.85 (0.73-0.98) |  |
| very much | 0.88 (0.67-1.16) |  |
|  |  |  |
| **Mental well-being and social relations** |  |  |
| Normal attitude towards food |  |  |
| yes | 1.00 |  |
| no | 0.80 (0.62-1,03) |  |
| cannot say | 0.76 (0.64-0.91) |  |
| Usual state of mind on scale -10 – +10 |  |  |
| high 8–10 | 1.00 | 1.00 |
| medium 0–7 | 0.90 (0.76-1.06) | 0.84 (0.70-1.00) |
| low -1 – -10 | 0.65 (0.52-0.82) | 0.64 (0.50-0.82) |
| Relationship with the parents on scale -10 – +10 |  |  |
| high 8–10 | 1.00 |  |
| medium 0–7 | 1.07 (0.93-1.24) |  |
| low -1 – -10 | 0.74 (0.56-0.97) |  |
| Experiencing various social situations (etc. giving presentation) on scale -10 –­­ +10 |  |  |
| high 8–10 | 1.00 |  |
| medium 0–7 | 0.87 (0.72-1.05) |  |
| low -1 – -10 | 0.82 (0.67-0.99) |  |
|  |  |  |
| **Other issues** |  |  |
| Presents willingness to discuss about sexual health |  |  |
| no | 1.00 | 1.00 |
| yes | 0.55 (0.46-0.66) | 0.67 (0.56-0.82) |
| Presents willingness to discuss about a non-specific matter |  |  |
| no | 1.00 | 1.00 |
| yes | 0.47 (0.41-0.55) | 0.55 (0.47-0.64) |
